# Women’s intentions to self-collect samples for human papillomavirus testing in an organized cervical cancer screening program

**DOI:** 10.1186/1471-2458-14-1060

**Published:** 2014-10-10

**Authors:** Laurie W Smith, Fareeza Khurshed, Dirk J van Niekerk, Mel Krajden, Sandra B Greene, Suzanne Hobbs, Andrew J Coldman, Eduardo L Franco, Gina S Ogilvie

**Affiliations:** British Columbia Cancer Agency, 711-750 West Broadway, Vancouver, BC V5Z 1H6 Canada; University of British Columbia, Vancouver, Canada; British Columbia Centre For Disease Control, Vancouver, Canada; University of North Carolina at Chapel Hill, North Carolina, USA; McGill University, Montreal, Canada

**Keywords:** Human papillomavirus (HPV), Cervical cancer screening, Self-collection, Intention, Theory of planned behaviour

## Abstract

**Background:**

Mounting evidence affirms HPV testing as an effective cervical cancer screening tool, and many organized screening programs are considering adopting it as primary testing. HPV self-collection has comparable sensitivity to clinician collected specimens and is considered a feasible option in hard-to-reach women. We explored women’s intentions to HPV self-collect for cervical cancer screening from a cohort participating in a Canadian randomized controlled cervical cancer screening trial.

**Methods:**

Women aged 25–65 were invited to complete an online survey assessing intentions to be screened with HPV testing instead of the Pap smear. The survey was based in the Theory of Planned Behaviour and questions were included to assess women’s intentions to self-collect for HPV. Demographic characteristics of women who intended to self-collect were compared with those who did not. Demographic and scale variables achieving a p-value <0.1 in the univariate and bivariate analyses were included in the stepwise logistic regression model. The final model was created to predict factors associated with women’s intentions to self-collect an HPV specimen for cervical cancer. Odds ratios were calculated with 95% confidence intervals to identify variables associated with a woman’s intention to self-collect for cervical cancer screening.

**Results:**

The overall survey response rate was 63.8% (981/1538) with 447 (45.6%) reporting they intended to self-collect, versus 534 (54.4%) reporting they did not. In the univariate analysis, women with more than high school education were more likely to self-collect. Women who intended to receive HPV testing versus the Pap smear were 1.94 times as likely to be in favour of self-collection and those who intended to self-collect had significantly higher attitudinal scores towards HPV self-collection. The adjusted odds ratio and 95% confidence interval from the multivariate analysis demonstrated attitude towards self-collection was the only significant variable predicting a woman’s intention to self-collect (OR 1.25; 95% CI: 1.22, 1.29).

**Conclusions:**

The primary predictor of a woman’s intention to HPV self-collect for cervical cancer screening was her attitude towards the procedure. From a program planning perspective, these results indicate that education and awareness may be significant contributing factors to improving acceptance of self-collection and subsequently, improving screening attendance rates.

**Electronic supplementary material:**

The online version of this article (doi:10.1186/1471-2458-14-1060) contains supplementary material, which is available to authorized users.

## Background

Over the past 50 years, screening with cytology (Pap smear) has significantly decreased cervical cancer incidence and mortality in countries where it has been practiced effectively [1, 2]. However, even where screening is widely available, irregular or non-attendance to cervical screening are significant barriers to further progress decreasing cervical cancer rates in high-risk women [3]. In high income countries, it is estimated that more than half of women found to have cervical cancer have a history of never, or infrequent screening [4, 5].

It is now well established that persistent infection with a high-risk genotype of the human papillomavirus (HPV) is necessary for the development of cervical cancer and its precursors [6, 7]. Mounting evidence confirms DNA testing for high-risk (hr)-HPV has higher sensitivity and negative predictive value for detection of cervical cancer or its precursors than cytology testing [8]. Given this knowledge, HPV testing is being considered for primary screening for cervical cancer in organized programs [9, 10]. The use of HPV self-sampling for hard to reach and under-screened populations with self-collection shows impressive sensitivity compared to clinician collected specimens for detecting high-grade lesions [11, 12]. For any number of reasons, women may not participate in cervical cancer screening (cultural, language, geographical and or access barriers for example) and self-collection offers an alternative to attending a visit with a clinician for screening. With self-collection, women insert a sampling device into the cervico-vaginal canal to collect the specimen themselves in a private setting. Specimens are then dropped off or mailed in for testing, thereby eliminating the need for a gynaecologic exam by a clinician. Studies evaluating women’s perceptions and uptake of self-sampling have found that women generally feel positively about performing the procedure [12–14].

In British Columbia (BC), cervical cancer screening is managed provincially, through a population based cervical cancer screening program. In the past 30 years, incidence and mortality rates for cervical cancer have declined and remain low, reflecting the impact of organized population-based screening [15]. In 2011, over 500,000 BC women received Pap tests through the program. Despite program success, of the 174 cases of invasive cervical cancers diagnosed in 2010, 42% of these women were screened more than 5 years ago or had no history of being screened [15]. Hysterectomy adjusted participation rates for women aged 20–69 years were 67.3% from 2009 to 2011 [15]. Studies have shown that self-sampling has the potential to increase participation rates in hard to reach and under-screened women in screening programs [8, 13, 16].

As organized settings begin to plan for the introduction of primary HPV testing for cervical cancer screening, it will be essential for program planners to address methods to improve participation for women who do not routinely attend for cervical cancer screening. The use of self-collection offers an important opportunity to improve uptake in non-attenders for screening. In this evaluation, we determined women’s intentions to self-collect an HPV specimen for cervical cancer screening in the setting of an HPV testing based screening program.

## Methods

### Participants

Study participants for this evaluation were recruited through the HPV FOCAL Study, a randomized controlled, three-armed trial conducted in British Columbia (ISRCTN79347302) [17, 18]. Approval to conduct the study was received from the BC Cancer Agency Research Ethics Board (REB) (REB approval: H06-04032) and all women in this evaluation consented to participate. Between January 2008 and March 2012, HPV FOCAL recruited over 25,000 BC women aged 25–65 years of age through the organized provincial cervical cancer screening program at the BC Cancer Agency. Upon exit from one of the study arms, women with email addresses were sent an invitation to complete the online web-based survey and if necessary, they were sent two additional invitation reminders to complete the survey. *FluidSurveys* (http://www.fluidsurveys.com) was the online survey software utilized for the purposes of this evaluation. Participants entered data on the website which was then downloaded into CSV files for analysis.

### Survey tool

The Theory of Planned Behaviour (TPB) [19, 20] was used as the theoretical framework for the survey in this study. This framework has been applied extensively to assess health behaviours and attendance at screening [21–23]. TPB proposes that the most important determinant of any behaviour is the person’s *intention* to perform that behaviour [21]. The Theory of Planned Behaviour is considered an important model of attitude-behaviour relationships, with the constructs of this framework showing to contribute to the prediction of intentions and subsequent behaviour [24]. All items included in the survey were constructed from literature review and feedback from content experts. The survey was reviewed by an expert in TPB and subsequently pilot tested on a small number of women in the target demographic after which it was revised and re-piloted again prior to implementation to eligible women.

At the beginning of the survey, women were provided with some brief background information on human papillomavirus, which included information on HPV prevalence, transmission, its role in cervical cancer and the reasons for use of HPV testing in cervical cancer screening (See Appendix 1). The survey assessed women’s intentions to be screened for HPV for cervical cancer instead of Pap smears; women’s intention to be screened for HPV at 4 year screening intervals, and; screening for HPV at 4 year intervals commencing at 25 years of age [25]. As part of the survey, women were informed that HPV specimens could be self-collected vaginally, without needing to see a health care provider or undergo a pelvic examination for cervical cancer screening. In addition to demographics, variables assessing the three specific elements that predict behaviour intentions were measured with seven point Likert scales. These included: attitude towards the behaviour, perceived behavioural control, and subjective norms to the behaviour. For the purposes of this analysis, “behaviour” refers to a woman’s willingness to collect her own sample for HPV testing. Attitudes towards the behaviour are one’s perspective on the value and utility of the behaviour. Perceived behavioural control refers to an individual’s perception of their ability to control the behaviour, and subjective norms to behaviour are one’s belief about how people they care about will view the behaviour in question (in other words, social pressure to perform or not perform the behaviour in question) [21]. Each variable can be measured either directly (asking about overall attitude), or indirectly (by asking respondents about specific beliefs about the behaviour), with both approaches to variable measurement make different assumptions about underlying cognitive structures [20].

### Response rate

Surveys were reviewed for completeness and where duplicate surveys were identified, the first complete survey was used in the analysis and the second was discarded. Response rate was calculated according to the American Association for Public Opinion Research [26]. Response rate for this survey was number of complete surveys, divided by number of complete surveys plus partially complete, refusal and log on without completion.

## Analysis

The primary endpoint for this evaluation was response to the statement ‘*I would be willing to collect my own sample/specimen for cervical cancer screening*’. Participants responded to the statement with a seven point Likert scale (strongly disagree, to strongly agree). The responses were dichotomized so that those who responded >4 were coded as ‘intending to self-collect’ and participants who responded ≤4 as ‘not intending to self-collect’. In addition, women’s attitudes towards self-collection were evaluated from responses to the statement ‘Collecting my own sample for cervical cancer screening would be… (accurate vs. inaccurate; safe vs. unsafe; protect my health vs. harm my health; acceptable vs. unacceptable)’.

Demographic characteristics of survey responders and non-responders were compared using data collected from the larger HPV FOCAL Trial. All FOCAL Trial participants are asked to complete a demographic questionnaire upon entry, with questions addressing such variables as marital status, ethnicity, smoking and sexual history. Descriptive and univariate analyses of demographic characteristics of survey respondents were performed, including median age, marital status, education, sexual history, ethnicity and smoking history, categorized by women who intend to self- collect, versus those who did not. Continuous variables were compared with Student’s t-tests and Kruskal-Wallis tests as appropriate and categorical variables were compared with Chi-Square test. Women’s intention to self-collect a sample for cervical cancer screening was calculated with 95% confidence intervals and variables with p-values <0.05 were deemed significant.

Scale items were analyzed according to methods for the *Theory of Planned Behaviour* [20]. See Additional file 1 for analysis details. In summary, items were re-anchored and re-coded as needed and if items in scales achieved agreement as measured by Cronbach’s alpha >0.5, a composite variable was created and then included in the univariate and where appropriate, multivariate analysis. If scales did not achieve a Cronbach’s alpha >0.5, subscales which did achieve agreement were created and included in analysis.

Demographic characteristics of women who intended to self-collect were compared with those who did not intend to self-collect with Chi-square, Student’s t-test and Kruskal-Wallis as appropriate. Multi-collinearity of psychological scales that achieved an item correlation with Cronbach’s alpha >0.5 was assessed with Pearson correlation coefficient. Overall scale scores and mean scores with standard deviations for scale results between those who intended to self-collect and those who did not intend to self-collect were calculated. Mean results with standard deviations between scales that had acceptable agreement (Cronbach’s alpha >0.5) and no collinearity were compared using Student’s t-tests. Demographic (including age, marital status, cultural background, number of male sexual partners, smoking history) and scale variables that achieved a p-value <0.1 in the univariate and bivariate analyses were included in the stepwise logistic regression model. The model was created to predict factors associated with women’s intentions to self-collect a specimen for cervical cancer. The dependent variable for the model was ‘intention to self-collect’ (0 = did not intend; 1 = intended). Logistic regression analysis was conducted and odds ratios calculated for significant variables with 95% confidence intervals to identify correlates of a woman’s intention to self-collect for cervical cancer screening. Analyses were conducted with SAS 9.3.

## Results

**Figure 1:**
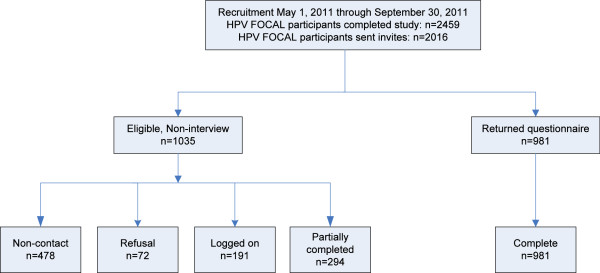
**Study Flowchart and participant distribution.**

The survey was administered from May through September 2011. Of the 2,459 women who had completed participation in the FOCAL trial at that time, email addresses were available for, and invitations to complete the survey were sent to 2,016 women (eligible population) (Figure 1). Of the 2,016 eligible women, 1,035 were not surveyed. This includes 191 who logged on but did not respond to any questions; 294 who submitted partially complete surveys; 478 emails were returned undeliverable and 72 responded to the invite, but declined participation. In total, 981 completed surveys are included in the analysis, for an overall response rate of 48.7% (981/2,016) for all women from the eligible population, and 63.8% (981/1,538) for all women who received the invite for survey completion.

The results demonstrated no significant differences between socio-demographic characteristics of survey responders and non-responders (Table 1). Survey respondents were between 25 and 65 years of age, with a mean age of 45.0 years (standard deviation [SD] 10.0). More than 85% of women self-reported having more than high school education of which 50% reported achievement of a University degree. The majority of responders, self-reported Caucasian, black or South Asian background (>89%); 2.4% of women were Aboriginal and 8.3% were Chinese. More than 56% reported having 5 or fewer lifetime sexual partners. Six percent were current smokers and 36.1% reported smoking at some time in their lives.

**Table 1:** **Comparison of demographic characteristics survey respondents vs. non-respondents** ^**1**^

Characteristic	Group	Study invitees N (%)	Respondent N (%)	Non-respondent N (%)	p-Value
Overall		2016	981	1035	
Age, Recruitment	Mean (SD)	45.1 (10.1)	45.0 (10.0)	45.3 (10.2)	0.5248
	Median (IQR)^2^	45.0 (38.0, 53.0)	45.0 (38.0,53.)	46.0 (37.0, 53.0)	
Education	Missing	130		130	0.2330
	<High School	31 (1.6%)	11 (1.1%)	20 (2.2%)	
	High School (Complete)	248 (13.1%)	122 (12.4%)	126 (13.9%)	
	Trade/College/ University (Incomplete)	692 (36.7%)	356 (36.3%)	336 (37.1%)	
	University graduate	584 (31.0%)	311 (31.7%)	273 (30.2%)	
	University advanced degree	331 (17.6%)	181 (18.5%)	150 (16.6%)	
Sexual partners - ever	Missing	151		151	0.8514
	0	4 (0.2%)	1 (0.1%)	3 (0.3%)	
	1	362 (19.4%)	185 (18.9%)	177 (20.0%)	
	2 to 5	693 (37.2%)	364 (37.1%)	329 (37.2%)	
	6 to 10	408 (21.9%)	221 (22.5%)	187 (21.2%)	
	11 to 50	376 (20.2%)	198 (20.2%)	178 (20.1%)	
	>50	22 (1.2%)	12 (1.2%)	10 (1.1%)	
Cultural background	Missing	128		128	0.2879
	Chinese	175 (9.3%)	81 (8.3%)	94 (10.4%)	
	Aboriginal	46 (2.4%)	24 (2.4%)	22 (2.4%)	
	Caucasian and other	1667 (88.3%)	876 (89.3%)	791 (87.2%)	
Smoke, Now	Missing	188		188	0.1908
	No	1707 (93.4%)	923 (94.1%)	784 (92.6%)	
	Yes	121 (6.6%)	58 (5.9%)	63 (7.4%)	
Smoke, Ever	Missing	184		184	0.4382
	No	1156 (63.1%)	627 (63.9%)	529 (62.2%)	
	Yes	676 (36.9%)	354 (36.1%)	322 (37.8%)	

^1^Pearson’s Chi Square, Student’s t-test; ^2^Kruskal-Wallis.

The Cronbach’s alpha was 0.96 (96%) indicating consistency of responses to the attitude measurements for self-collection.

Of the 981 women who completed the surveys, 447 (45.6%) reported they intended to self-collect, versus 534 (54.4%) who reported they did not intend to self-collect (Table 2). Women who had more than high school education were more likely to self-collect than women with less than high school education (p-value =0.02). There were no significant differences between marital status, sexual history, ethnic origin, smoking status age of recruitment of women who intended to self-collect vs. those who did not (p-value >0.05).

**Table 2:** **Univariate demographic characteristics of women who intend to self-collect compared to those who do not** ^**1**^

Variable	Group	SC25 < =4 Do not intend to self-collect	SC25 > 4 Intend to self-collect	Overall	p-value
Overall	Overall	534 (54.4%)	447 (45.6%)	981	0.0055
Marital status	Divorced	56 (10.5%)	52 (11.6%)	108 (11.0%)	0.5366
	Married	317 (59.4%)	281 (62.9%)	598 (61.0%)	
	Never married	69 (12.9%)	43 (9.6%)	112 (11.4%)	
	Widowed	5 (0.9%)	2 (0.4%)	7 (0.7%)	
	Common-law	50 (9.4%)	41 (9.2%)	91 (9.3%)	
	Did not answer	37 (6.9%)	28 (6.3%)	65 (6.6%)	
Education	<High School	85 (15.9%)	48 (10.7%)	133 (13.6%)	0.0193
	More than High School	499 (84.1%)	399 (89.3%)	848 (86.4%)	
Sexual partners	0	0 (0.0%)	1 (0.2%)	1 (0.1%)	0.1908
	1	116 (21.7%)	69 (15.4%)	185 (18.9%)	
	2-5	189 (35.4%)	175 (39.1%)	364 (37.1%)	
	6-10	116 (21.7%)	105 (23.5%)	221 (22.5%)	
	11-50	105 (19.7%)	93 (20.8%)	198 (20.2%)	
	51-99	7 (1.3%)	3 (0.7%)	10 (1.0%)	
	>99	1 (0.2%)	1 (0.2%)	2 (0.2%)	
Ethnic origin	Chinese	50 (9.4%)	31 (6.9%)	81 (8.3%)	0.3876
	Aboriginal	13 (2.4%)	11 (2.5%)	24 (2.4%)	
	Other	471 (88.2%)	405 (90.6%)	876 (89.3%)	
Smoke - Ever	No	339 (63.5%)	288 (64.4%)	627 (63.9%)	0.7585
	Yes	195 (36.5%)	159 (35.6%)	354 (36.1%)	
Age (Recruitment)	Median (IQR)^2^	44 (37–52)	46 (38–54)	45 (38–53)	0.0927

^1^Chi Square test. ^2^Kruskal-Wallis.

A woman’s responses to other questions in the survey can be directly correlated with her opinions regarding self-sampling. Variables that have a significant difference in scores between those who intend to self-collect and those who do not, include subjective norms indirect (p-value = 0.03) and direct (p-value <0.01), which may indicate that the opinions of those who are important to them, as well as others such as family physician, friends, partner, the BC Cancer Agency, may influence the woman’s decision to self-collect or not (Table 3). Women who intended to self-collect had significantly higher attitudinal scores, indicating belief that self-collection was accurate, safe, protective and acceptable (p-value < 0.001). There was no difference between women who intended to self-collect and those who did not with regards to comfort sharing results with partners (p-value = 0.28) or reported perceived behavioural control (p-value = 0.10).

**Table 3:** **Comparison of scale results between women intending versus not intending to self-collect**

Variable	Overall (SD)	Do not intend to self-collect SC25 < =4 Mean (SD)	Intend to self-collect SC25 > 4 Mean (SD)	P-Value (Student’s t-test)
Subjective norms indirect	34.79 (31.93)	32.71 (32.36)	37.27 (31.25)	0.0255
Age	44.96 (9.99)	44.49 (9.93)	45.52 (10.03)	0.1078
Contacting partners	12.59 (2.25)	12.52 (2.33)	12.68 (2.14)	0.2781
Perceived behavioural control	23.41 (4.12)	23.21 (4.04)	23.65 (4.21)	0.0950
Attitude towards self-collection	17.15 (7.62)	12.91 (6.30)	22.23 (5.72)	<.0001
Subjective norms	11.01 (2.57)	10.78 (2.61)	11.30 (2.49)	0.0014

We also examined the relationship between a woman’s intent to self-collect, and her intent to receive HPV testing versus the Pap smear. Those who intend to receive HPV testing were 1.94 times as likely to be in favour of self-collection (95% CI: 1.35; 2.80, p = 0.0003) than women who did not intend to receive HPV testing.

Based on the univariate and bivariate analyses, variables included in the regression model were those with a p-value less than 0.1 including: the psychological variables for indirect and direct subjective norms; perceived behavioural control; intent to receive HPV testing versus the Pap smear; and attitude towards self-collection. Age and education were also included in the model. We used a stepwise regression analysis that did not retain insignificant explanatory variables. The only variable of significance in predicting a woman’s intention to self-collect a cervical specimen was her attitude towards self-collection (OR 1.25; 95% CI: 1.22, 1.29) (Table 4).

**Table 4:** **Predictors of Intention to self-collect for cervical cancer screening with HPV testing using logistic regression**

Variable	Odds ratio	95% Confidence limits	
Attitudes to self-collection (SC24)	1.254	1.220	1.289

## Discussion

It is anticipated that vaccination against oncogenic HPV types will decrease the incidence of cervical cancer on a population level in the future when girls who have been vaccinated begin to reach the age for cervical cancer screening sometime in the next 10–15 years. However, despite the promising effects widespread HPV vaccination will have, screening for cervical cancer will still need to occur. With the growing body of evidence demonstrating the efficacy of primary HPV testing with cytology triage for detecting precancerous lesions and subsequent decreases in the incidence of cervical cancer [27], it is anticipated programmatic changes will occur in many organized settings from primary screening with the Pap smear, to HPV testing. However, a paradigm shift to more sensitive technologies will not necessarily improve attendance rates for under-screened women. In order to decrease population incidence of cervical cancer and pre-cancerous lesions in vaccinated and or unvaccinated cohorts, improved screening attendance rates must be realized. Therefore, alternative methods such as self-collection for HPV are being explored to improve participation rates. Prior to implementation of this approach, a component of program planning should include examination of women’s acceptance of, and willingness to participate in screening with HPV self-collection in the organized setting, as well as healthcare provider and population education.

In our study, of the 981 women who completed the survey, 447 (45.6%) indicated they intended to self-collect versus 534 (54.4%) reporting they did not intend to self-collect. Overall, the results of this analysis show that regardless of other variables, the primary predictor of a woman’s intention to HPV self-collect for cervical cancer screening is her attitude towards the procedure. This is important from a program planning perspective, indicating that concerted efforts towards comprehensive education and awareness campaigns on the value of self- collection and HPV testing in preventing cancer could be the significant contributing factors to improving acceptance of self-collection and subsequently improving screening attendance rates.

In the current study, when asked if they would be willing to collect their own specimen for HPV, more than half of all responders were not willing to self-collect. This rate was higher than expected, given results of previously published literature which found that women generally approved of the procedure and had positive attitudes towards it [12, 14, 28]. There are several potential reasons for this. Women in our study are currently actively engaged in cervical cancer screening, and thus are very comfortable and even prefer having a clinician collect their specimens for screening. This is important, because if self-collection is used as part of a regular screening program, our findings indicate need for substantial education and support for women already engaged in screening. In addition, in our study, we explored women’s willingness to self-sample through the survey and did not provide an extensive description about how the procedure is performed, nor were women provided the opportunity to self-collect. In our previous study, Ogilvie et al., [25] found that among the predictors of intention to be screened with HPV were positive attitudes towards HPV and recommendations for HPV testing from highly regarded health agencies or health practitioners. The results of this evaluation correlate with those findings, indicating that providing women with sufficient information and education about HPV self-collection is a significant factor contributing to willingness to perform the procedure. The authors recommended that substantial efforts should be made to ensure women are educated about the safety and accuracy of HPV testing as these are critical factors to women’s acceptance of HPV testing [25].

In this study, the women who intended to be screened with primary HPV testing compared to the Pap smear were significantly more likely to be in favour of self-collection. This is relevant, given our previous study showed that 84.2% of women intended to be screened for cervical cancer with HPV testing versus the Pap test [25]. However, our previous study also showed that intention to be screened with HPV decreased to 54.2% when the interval was extended to every 4 years and decreased further to 51.4% when HPV testing every 4 years commencing at age 25 was proposed [25].

The results of the descriptive analysis showed women who had more than high school education were more likely to self-collect than women with less than high school education. However, education was not significant in the adjusted regression model and the only predictor of significance was a woman’s attitude towards self-collection. This is consistent with other published research demonstrating no definitive associations between a woman’s attitude towards self-collection and demographic variables such as education, socioeconomic status and age [12, 29]. Historically, under-screened women in organized programs are often from lower socioeconomic status and/or have lower education levels [3, 30]. This finding is important and suggests that regardless of socioeconomic factors, efforts made to address women’s attitudes towards self-sampling are critical to successful implementation of the approach. From a program planning perspective, adoption of a variety of appropriate knowledge translation activities will be critical to successful adoption of alternative approaches to screening and improved attendance rates. This survey was administered to women who are attendees of the British Columbia, organized cervical cancer screening program and participants of a randomized controlled trial (RCT) [17, 18]. All women surveyed had a recent cervical screen (less than 3 years) demonstrating a level of comfort receiving screening from a clinician. This indicates that these survey results are highly generalizable for screening programs looking to broadly adopt self-collection, and offer it to women as an option for cervical cancer screening. Although the comparison of results between survey respondents and non-respondents showed there were no significant demographic differences, because our survey primarily included women engaged in a screening program, the findings of the survey can only be generalized with caution to the population of women who do not routinely attend for cervical cancer screening. Over 50% of our survey participants reported having a university degree, and 89% reported Caucasian/other which may not necessarily reflect the target population for whom self-collection may initially be offered. In addition, this study was conducted in an urban setting, with less representation of women from rural or remote settings. Given self-collection for cervical cancer screening is also considered to be an option to improve rates in the under-screened, who often have lower education are from ethnic minorities, and or are from rural/remote settings, further examinations need to be targeted to these populations specifically.

To our knowledge, women in this survey had no experience performing cervical sample self-collection. Other trials demonstrating positive attitudes towards self-collection assessed women’s attitudes towards the procedure after having obtained a self-collected specimen [12, 14]. A recent meta-analysis examining published research comparing self-collected HPV testing in women who did not routinely participate in screening programs showed high acceptance levels for self-collection in this population [14] demonstrating the importance of specifically targeting the under-screened and providing the opportunity to perform the procedure in explorations of self-collection as an alternative method in organized settings. Further research is recommended to specifically target the under-screened in British Columbia with the opportunity to participate in self-collection procedures to evaluate not only attitudes and acceptance, but also screening participation rates.

## Conclusion

This study demonstrates that the primary predictor of a woman’s intent to self-collect a vaginal sample for HPV testing is her attitude towards this procedure. Although the women in this study are currently engaged in clinician-based screening in an organized program, these findings illustrate that comprehensive education is essential for successful implementation of self-collection to both keep women engaged in screening and to improve screening attendance rates. Culturally competent educational materials should be available in a variety of formats to include information about HPV, its association with cervical cancer, and the importance of screening. Additionally, to ensure acceptance of self-collection by women, they should be provided with information regarding the ease, effectiveness and safety of the procedure. Further research is recommended to explore the attitudes surrounding, and intentions to self-collect, for women who do not regularly attend for clinician-based cervical cancer screening.

## Appendix 1

Introductory information provided to survey respondents

*Here is some background information for you to consider before you complete this survey.*

*The human papillomavirus (HPV) is a common virus that can infect the cervix (part of a woman’s womb). It is now known to be the cause of cervical cancer. Women develop HPV infections in the cervix after having sexual activity with a partner who is infected with HPV. However, HPV is so common that over 75% of sexually-active women will have an HPV infection of their cervix sometime during their life. Most women who find out they have an HPV infection in the cervix after the age of 30, were infected with HPV years before. Over 90% of women who are infected with HPV in the cervix get rid of the infection naturally. It is only women who have longstanding infections with certain types of HPV who may be at risk for developing cervical cancer. Women may not have known it in the past, but it is these same HPV infections that are the most common reason for abnormal Pap smears.*

*Right now in BC, women start cervical cancer screening once they become sexually active. We now know that testing for HPV infections in the cervix is more accurate than the Pap smear for predicting whether or not a woman will develop cervical cancer.*

## Authors’ information

- LWS MPH BN RN: Project Manager, HPV FOCAL Study, Population Oncology, BC Cancer Agency (BCCA). Vancouver, Canada.
- FK MSc in Statistics: No current affiliation.
- DJV, FRCP(C): Medical Leader BC Cervical Cancer Screening Program, BCCA, Vancouver, Canada
- MK, MD FRCP(C): Medical Head Hepatitis Clinical Prevention Services, Associate Medical Director, BC Centre for Disease Control (BCCDC) Public Health Microbiology and Reference Laboratory. Professor Dept. of Pathology and Laboratory Medicine, University of British Columbia (UBC). Vancouver, Canada.
- SBG DrPH: Professor of the Practice and Interim Chair, Health Policy and Management,

Gillings School of Global Public Health. University of North Carolina (UNC) at Chapel Hill. North Carolina, USA.

- SH DrPH: Clinical Associate Professor, Director, Doctoral Program in Health Leadership.

Research Fellow, Center for Health Promotion and Disease Prevention. Department of Health Policy and Management, and Department of Nutrition, Gillings School of Global Public Health.

UNC at Chapel Hill. North Carolina, USA.

- AJC PhD: Vice President, Population Oncology, BCCA. Adjunct Professor Statistics, UBC. Vancouver, Canada.
- ELF, DrPH FRSC FCAHS: James McGill Professor in the Departments of Oncology and Epidemiology & Biostatistics, Director, Division of Cancer Epidemiology, and Chair, Department of Oncology, at McGill University. Fellow of the Canadian Academy of Health Sciences and of the Royal Society of Canada. Editor-in-Chief of Preventive Medicine. Montreal, Canada
- GSO, MD MSc FCFP DrPH: Medical Director Clinical Prevention Services, BCCDC. Associate Professor, Faculty of Medicine, UBC. Vancouver, Canada.

## Electronic supplementary material

Additional file 1: **Theory of Planned Behaviour.** (DOC 104 KB)

## Abbreviations

HPV: Human papillomavirus
BC: British Columbia
TPB: Theory of planned behaviour
RCT: Randomized controlled trial
REB: Research ethics board.
