# Supplementary material for: Women’s intentions to self-collect samples for human papillomavirus testing in an organized cervical cancer screening program
Source: BMC Public Health. 2014 Oct 10;14:1060. doi: 10.1186/1471-2458-14-1060 (PMC4203923; doi:10.1186/1471-2458-14-1060)
Supplement: Supplementary file 1 — Additional file 1: Theory of Planned Behaviour. (DOC 104 KB) [file 12889_2014_7163_MOESM1_ESM.doc]

**Additional file 1**

**APPENDIX 2**:

**Theory of Planned Behaviour**


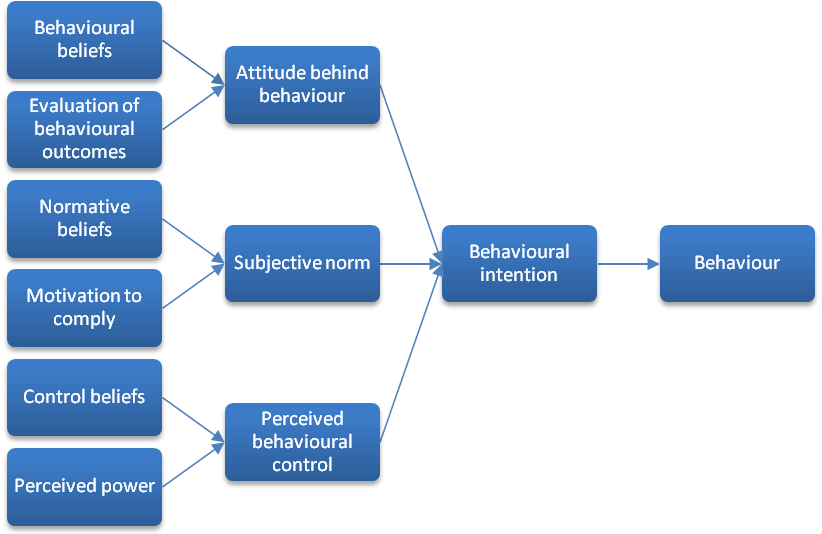


TPB’s explanation for how *behavioural intention* determines *behaviour*, and how *attitude toward behaviour*, *subjective norm*, and *perceived behavioural control* influence *behavioural intention*. According to the model, attitudes toward behaviour are shaped by beliefs about what is entailed in performing the behaviour and outcomes of the behaviour. Beliefs about social standards and motivation to comply with those norms affect *subjective norms*. The presence or lack of things that will make it easier or harder to perform the behaviour affect *perceived behavioural control*. Thus, a causal chain of beliefs, attitudes, and intentions drives *behaviour*.

*Source:* [*http://www.cancer.gov/cancertopics/cancerlibrary/theory.pdf*](http://www.cancer.gov/cancertopics/cancerlibrary/theory.pdf)*, accessed on September 8, 2014*

Survey questions were designed according to the Theory of Planned Behaviour (TPB). Multiple questions were designed for each section and then aggregated according to a pre-determined analysis plan. All questions in the survey were on a seven point Likert scale. The analysis plan that was used for this analysis follows.

**HPV ACCEPTABILITY ANALYSIS PLAN**

*Study Measures:* Measures were derived from a comprehensive literature review, elicitation interviews with women and consultation with an expert in TPB.  A pilot survey was drafted and revised based on feedback.  All items used a seven point Likert scale. Overall attitude to having an HPV test instead of Pap screening will be assessed with four items which will be summed to form an attitude scale.  Attitudes to having an HPV test instead of Pap screening will also be assessed in the context of an extended screening interval and in the context of an extended screening interval and delayed start at the age of 25. All scales were anchored in the same direction, so no recoding will be needed.  Item analysis using Cronbach’s α will be conducted to determine internal consistency. Assuming internal consistency is >0.5, a composite variable will be created for the analysis.

For direct measures of subjective norms, 3 items will be assessed for consistency, and summed if Cronbach’s α is >0.5. To create the normative belief score for the model for indirect norms, the belief score is multiplied by the score for motivation to participate in the named activity. For indirect norms, the Likert scales of 1-7 that measure normative belief (Family Physician, Friends, Spouse/Partner, BC Cancer Agency) will be re-coded to a scale of -3 to +3. With this recoding, positive score indicates that overall, the individual experiences pressure from the individual/group named in the item to participate in the activity. To then create item, the normative belief is multiplied by motivation to comply, thus creating 4 indirect measures.  The internal consistency of indirect items will then be assessed by Cronbach’s alpha.  In the case of indirect norms, if there is low internal consistency, each item will be examined individually, and subscales based on combinations of 2 or 3 of the indirect items will be created.  This is because the indirect norm of family physician influence may not necessarily be correlated with that of the influence of a friend or spouse. Based on these analyses, either a combined scale or individual items will finalized for the model.

Perceived behavioural control consists of 4 items and will be assessed for consistency.  If they have low internal consistency, we will re-examine to create subscales, likely based on self efficacy and control.  Based on these evaluations, items will be summed, either as two subscales or as an overall scale for PBC.

Along with the traditionally used TPB measures, a variable for ‘contacting partners’ was created for this analysis. One of the unique aspects of cervical cancer screening with HPV is that HPV is a communicable disease, and thus there may be an expectation that sexual partners should be advised of an individual’s HPV status.  This may influence a woman’s willingness to participate in cervical cancer screening, as she may ultimately need to discuss a communicable disease with her partner. Two items assessing the impact of needing to let a partner know about HPV status on decision to receive screening were included, in order to determine whether the communicable disease/STI element of HPV would at all impact on willingness to have HPV testing.  These two items will be assessed for internal consistency and if high, summed and included as an independent variable in the analysis.
